# Cytotoxic Interactions Between Cannabidiol and Ionophore Coccidiostats in Feline and Canine Kidney Cell Lines—An In Vitro Study

**DOI:** 10.3390/molecules31213810

**Published:** 2026-10-22

**Authors:** Lidia Radko, Oliwia Kończak, Ovinuchi Ejiohuo, Maciej Gogulski, Joanna Zeyland

**Affiliations:** 1 Department of Preclinical Sciences and Infectious Diseases, Faculty of Veterinary Medicine and Animal Sciences, Poznan University of Life Sciences, 60-637 Poznan, Poland; maciej.gogulski@up.poznan.pl; 2 Experimental Center, National Veterinary Research Institute, 24-100 Pulawy, Poland; 3 Department of Biochemistry and Biotechnology, Poznan University of Life Sciences, 60-632 Poznan, Poland; oliwia.konczak@up.poznan.pl (O.K.); ovinuchi.ejiohuo@up.poznan.pl (O.E.)

**Keywords:** ionophore coccidiostats, cannabidiol, cytotoxicity, interaction, dog, cat, renal cell lines

## Abstract

The use of ionophore coccidiostats—salinomycin (SAL), lasalocid (LAS), monensin (MON), and narasin (NAR)—in animal production raises toxicity concerns in non-target species through feed contamination and because of their narrow safety margins. Cannabidiol (CBD) has antioxidant and cytoprotective properties that may modify the cellular response to ionophore-induced toxicity. This study examined the effect of CBD on the cytotoxicity of SAL, LAS, MON, and NAR in feline (CRFK) and canine (MDCK) kidney cells. Cells were exposed for 72 h to each coccidiostat, alone or in combination with CBD, and assessed using mitochondrial activity (MTT), lysosomal activity (NRU), total protein content (TPC), and membrane integrity (LDH) assays; the interaction was evaluated by isobolographic analysis. Molecular docking of CBD and the ionophores to human VDAC1 (PDB 6TIQ) was performed in AutoDock Vina 1.2.7, both individually and in simultaneous multi-ligand combinations, with β-NADH, VBIT-4, and SW016789 as reference ligands. All four ionophores induced concentration-dependent cytotoxicity, with distinct, compound- and species-specific profiles between CRFK and MDCK cells. CBD co-exposure produced divergent interactions depending on the drug and cell model, ranging from marked cytoprotection to pronounced synergistic potentiation of toxicity, most notably for SAL and LAS in canine cells. The ionophores occupied the N-terminal helix, and cannabidiol occupied a partially overlapping C-terminal region, from which β-NADH displaced it, suggesting a plausible structural basis for the observed interactions. These findings indicate that ionophore nephrotoxic potential and its modulation by CBD are both concentration- and species-dependent, warranting further investigation using additional in vitro and in vivo models.

## 1. Introduction

The presence of food and feed contaminants represents a significant challenge in ensuring the health and safety of both humans and animals, including companion animals (dogs and cats). Official monitoring of livestock feed effectively mitigates and detects existing hazards [1]. However, official surveillance of companion animal pet food worldwide requires stricter enforcement and harmonization to prevent potential contamination with veterinary drugs, metals, pesticides, and other substances. Particular concern surrounds the presence of veterinary medicinal products utilized in food-producing animals, including ionophore coccidiostats (salinomycin, lasalocid, monensin, and narasin), whose narrow safety margin poses a high risk of poisoning in non-target animals [2,3,4].

Numerous cases of coccidiostat poisoning in dogs and cats have been documented in the literature [5,6,7,8,9,10,11,12,13]. Among the clinical signs of poisoning, cardiac and skeletal muscle toxicity (myodegeneration, rhabdomyolysis) is the most consistently reported finding; renal dysfunction has also been described, arising both directly and secondarily to muscle damage [13]. Polyuria, followed by oliguria and subsequent renal failure, has been observed. Concurrently, histopathological changes—including bilateral renal medullary necrosis, fibrosis, mineralization of tubules and glomeruli, loss of nephrons, and medullary scarring, often accompanying concurrent skeletal muscle degeneration—have been reported in dogs fed coccidiostat-contaminated food [3,5,6,9]. These alterations were accompanied by high concentrations of the detected coccidiostats within the kidneys. Due to the mechanism of action of coccidiostats, an influx of Na^+^, K^+^, Ca^2+^, and H^+^ ions into cardiac, skeletal muscle, and renal tubular cells occurs, leading to mitochondrial swelling, loss of membrane potential, and a drastic decline in ATP levels. Consequently, renal damage may result both directly and secondarily—through myoglobinuric nephropathy following rhabdomyolysis and through hemodynamic (prerenal) disturbances [5,8,9,11,12].

In recent years, the production of animal products containing hemp extracts has increased, gaining substantial popularity. Among the bioactive compounds, cannabidiol (CBD) attracts particular attention, as it is a non-psychoactive phytocannabinoid possessing antioxidant and anti-inflammatory properties [14,15,16,17,18,19,20,21,22,23,24,25,26,27]. The multiple beneficial effects are due to the pleiotropic mechanisms of CBD. One of these mechanisms of CBD is the reduction in oxidative stress conditions and the reduction in reactive oxygen and nitrogen species (ROS/RNS) production. Among others, superoxide anion levels are reduced due to increased superoxide dismutase (SOD) and glutathione peroxidase induced by CBD. Other antioxidant enzymes, such as catalase (CAT), which catalyzes the conversion of H_2_O_2_ to water, may protect cells from oxidative stress [16]. A second protective mechanism of CBD is the reduction in the expression of inflammatory cytokines such as IL-6 and IFN-α and the production of matrix metallopeptidases (MMPs) [14]. CBD also plays an important role in maintaining ionic balance during stressful conditions; for example, it can modulate the Na^+^/Ca^2+^ exchanger and promote Ca^2+^ storage. CBD administration also inhibits apoptosis and supports cell survival. During cell survival, upregulated HO-1 may play a key role [17]. Numerous reports show that, unlike other cannabinoids, CBD has weak effects on cannabinoid receptors [18]. However, CBD activates transient receptor potential (TRP) channels such as TRPA1, TRPV1, and TRPV4, even at nanomolar concentrations [19].

Treatment of mice with cannabidiol significantly attenuated cisplatin-induced oxidative/nitrosative stress, inflammation, and cell death in the kidneys and improved renal function [20]. The related CBD suppressed inflammatory cytokine mRNA in cisplatin-induced nephropathy and significantly reduced apoptosis by inhibiting caspase-3 activity [21]. The effect of CBD on drug-induced nephropathies is the current subject of many studies to determine the mechanism of action of CBD [22].

Due to its cellular mechanism of action (mitochondrial functioning and regulation of intracellular Ca^2+^ concentration), it may modulate the nephrotoxicity induced by ionophore coccidiostats [14,15,16,17,18,19,20,21,22,23,24]. Recent studies indicate a nephroprotective effect of CBD against veterinary drugs, including ionophore coccidiostat cytotoxicity in human embryonic kidney cells (HEK-293) [25,26,27]. However, evaluating the nature of interactions between coccidiostats and CBD in dogs and cats requires experimental assessment at the cellular level using species-specific cell models.

The use of Crandell-Rees Feline Kidney (CRFK) and Madin-Darby Canine Kidney (MDCK) cells to study coccidiostat nephrotoxicity and its interaction with CBD is justified by the physiological role of the kidney in the elimination of these compounds, as well as by clinical and pathological evidence of renal involvement in poisoning cases [2,3,5,8,9,10,11,12,13]. In cats, salinomycin poisoning has most often been associated with a peripheral polyneuropathy rather than overt renal failure [6,9]. Nevertheless, given the shared ionophore mechanism of action and the central role of the kidney in drug elimination in both species, feline renal susceptibility to coccidiostats warrants direct experimental evaluation.

This study examined the influence of cannabidiol on coccidiostat-induced nephrotoxicity across two renal cell models: cat (CRFK) and dog (MDCK) cell cultures. Over a 72 h exposure period, cells were treated with the studied coccidiostats alone (concentrations: 1.0, 5.0, 10.0, and 50.0 µg/mL) or in combination with CBD at 1.56 µg/mL, a concentration that did not impair mitochondrial activity or membrane integrity in either cell line. Cytotoxic responses were characterized by a battery of assays measuring mitochondrial (MTT assay) and lysosomal (NRU assay) function, proliferative capacity (TPC assay), and cellular membrane damage (LDH assay). From these data, IC_50_ values were determined for the coccidiostats studied when administered both individually and alongside CBD. Additionally, the drug–CBD interactions were characterized quantitatively using the combination index (CI) approach. To complement the in vitro findings and elucidate the underlying structural mechanisms, molecular docking of CBD and ionophores to human VDAC1 (PDB 6TIQ) was conducted in AutoDock Vina 1.2.7, using β-NADH, VBIT-4, and SW016789 as reference ligands.

## 2. Results

### 2.1. The Effects of CBD in Feline Kidney (CRFK) and Dog (MDCK) Cells

Cannabidiol alone was tested over the range 1.56–12.5 µg/mL in both cell lines to establish its intrinsic activity (Table 1, Figure S1). In CRFK cells, NRU fell to 67.5% of the negative control at 1.56 µg/mL and to 29.4% at 12.5 µg/mL, and TPC fell from 89.5% to 66.6%, with significant inhibition at every concentration. MTT declined only at 12.5 µg/mL (86.3%), and LDH release did not exceed 3.1%. In MDCK cells, MTT increased to 117.5% of the negative control at 1.56 µg/mL, and TPC increased to 107.7%. Significant inhibition occurred only at 12.5 µg/mL, where NRU fell to 80.3% and total protein content to 91.7%. LDH release did not exceed 7.0%.

### 2.2. The Effects of Salinomycin and Its Interaction with CBD in Feline Kidney (CRFK) and Dog (MDCK) Cells

**Figure 1:**
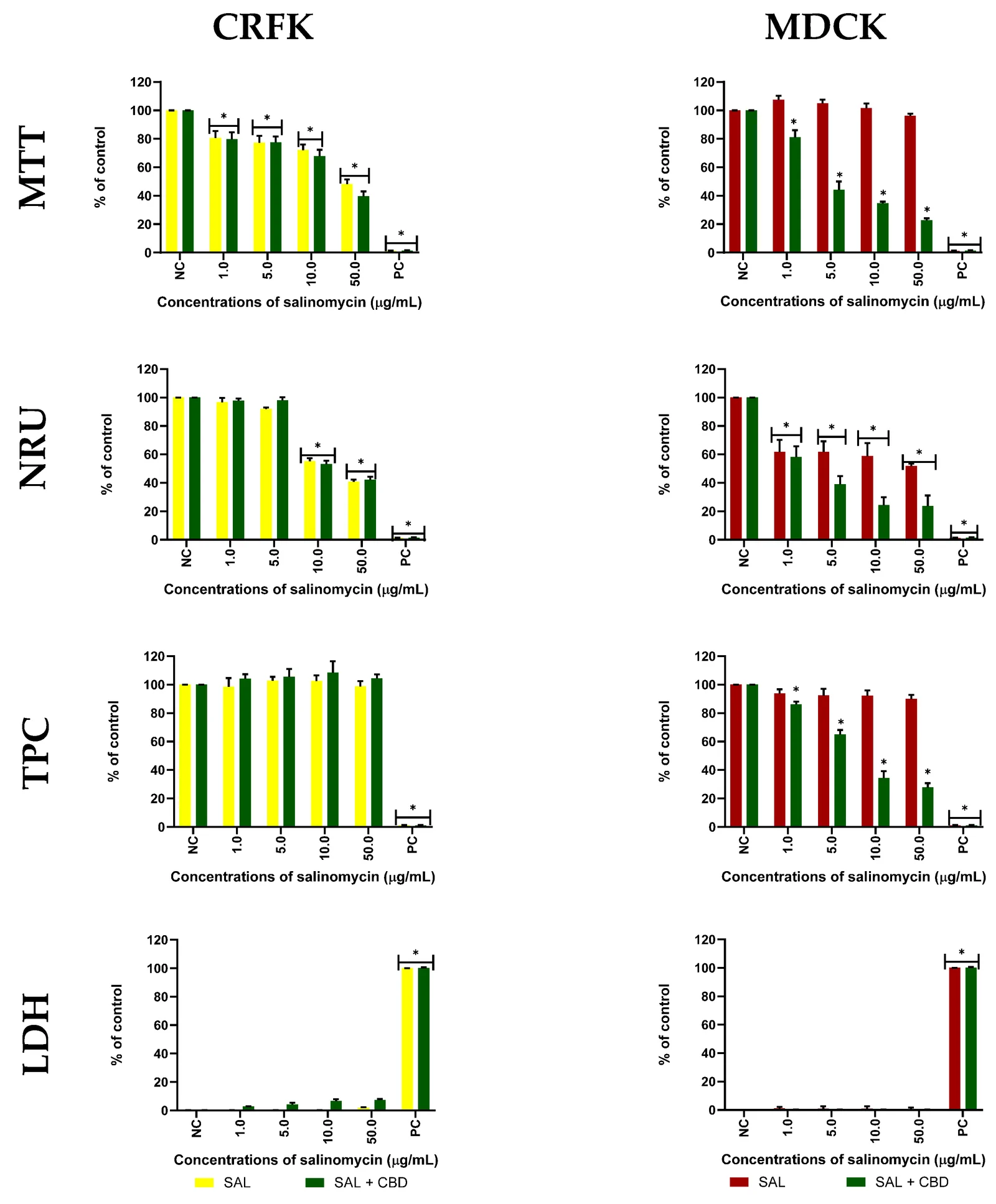
The inhibitory effect of salinomycin (SAL) and its interaction with cannabidiol (CBD) at concentrations of 1.56 µg/mL are shown in two kidney cell lines from cat (CRFK) and dog (MDCK). The study was performed using four assessment tests: mitochondrial activity (MTT assay), lysosomal activity (NRU assay), proliferation (TPC assay), and cell membrane integrity (LDH assay). Data are presented as mean ± SD (standard deviation), *n* = 3. NC—negative control (0.1% DMSO in medium for SAL or 0.2% DMSO in medium for SAL + CBD); PC—positive control (1% Triton X-100 in medium). * *p* ≤ 0.05.

Salinomycin inhibited mitochondrial and lysosomal activity in CRFK cells, starting at concentrations of 1.0 and 10.0 µg/mL, respectively (*p* ≤ 0.05) (Figure 1). The presence of CBD increased the drug concentration required to inhibit the assessed endpoints.

In CRFK cells, the lowest IC_50_ values for the drug and its mixture with CBD were observed in the MTT assay compared to the NRU assay (Table 2). However, the IC_50_ values for the mixture in both assays were higher compared to the effect of the drug alone.

**Figure 2:**
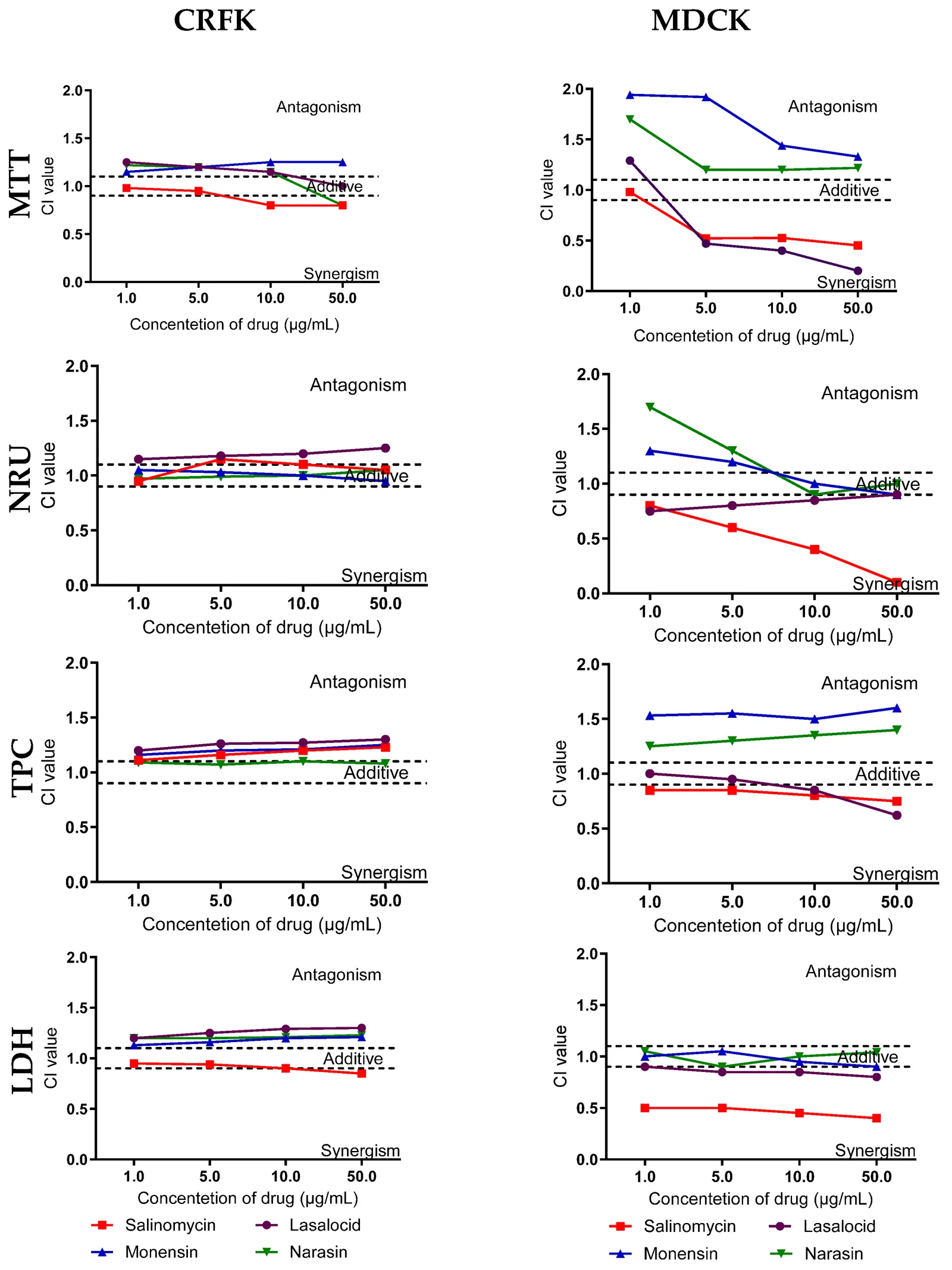
Combination index–fraction affected curves (CI plot) for the combination of four coccidiostats with cannabidiol at concentration of 1.56 µg/mL in CRFK and MDCK cells, using four different assays. Vertical bars represent 95% confidence intervals for CI values based on sequential deletion analysis. Horizontal dashed lines indicate the additive threshold, separating synergistic and antagonistic interactions.

An additive nature of the interaction between SAL and CBD was observed in the inhibition of lysosomal activity of cells (NRU; antagonistic only at 5 µg/mL) and in the disruption of cell membrane integrity (LDH) up to 10 µg/mL, becoming synergistic at 50 µg/mL. However, a weakly antagonistic interaction was observed in the inhibition of cell proliferation (TPC). In the MTT assay, the interaction was additive at 1 and 5 µg/mL and synergistic at high drug concentrations (10 and 50 µg/mL) (Figure 2, Table 3).

The effect of salinomycin on MDCK cells differed from that observed in CRFK cells. In MDCK cells, salinomycin inhibited lysosomal activity starting at the lowest concentration tested, 1 µg/mL (Figure 1). Co-treatment with CBD resulted in decreased lysosomal and mitochondrial activity and cell proliferation at a concentration as low as 1 µg/mL (Figure 1). This resulted in a lower IC_50_ value for the mixture compared to the drug alone (Table 2). Analysis of the interaction demonstrated a synergistic effect, indicating that CBD potentiated the cytotoxicity of salinomycin in MDCK cells (Figure 2).

### 2.3. The Effects of Lasalocid and Its Interaction with CBD in Feline Kidney (CRFK) and Dog (MDCK) Cells

**Figure 3:**
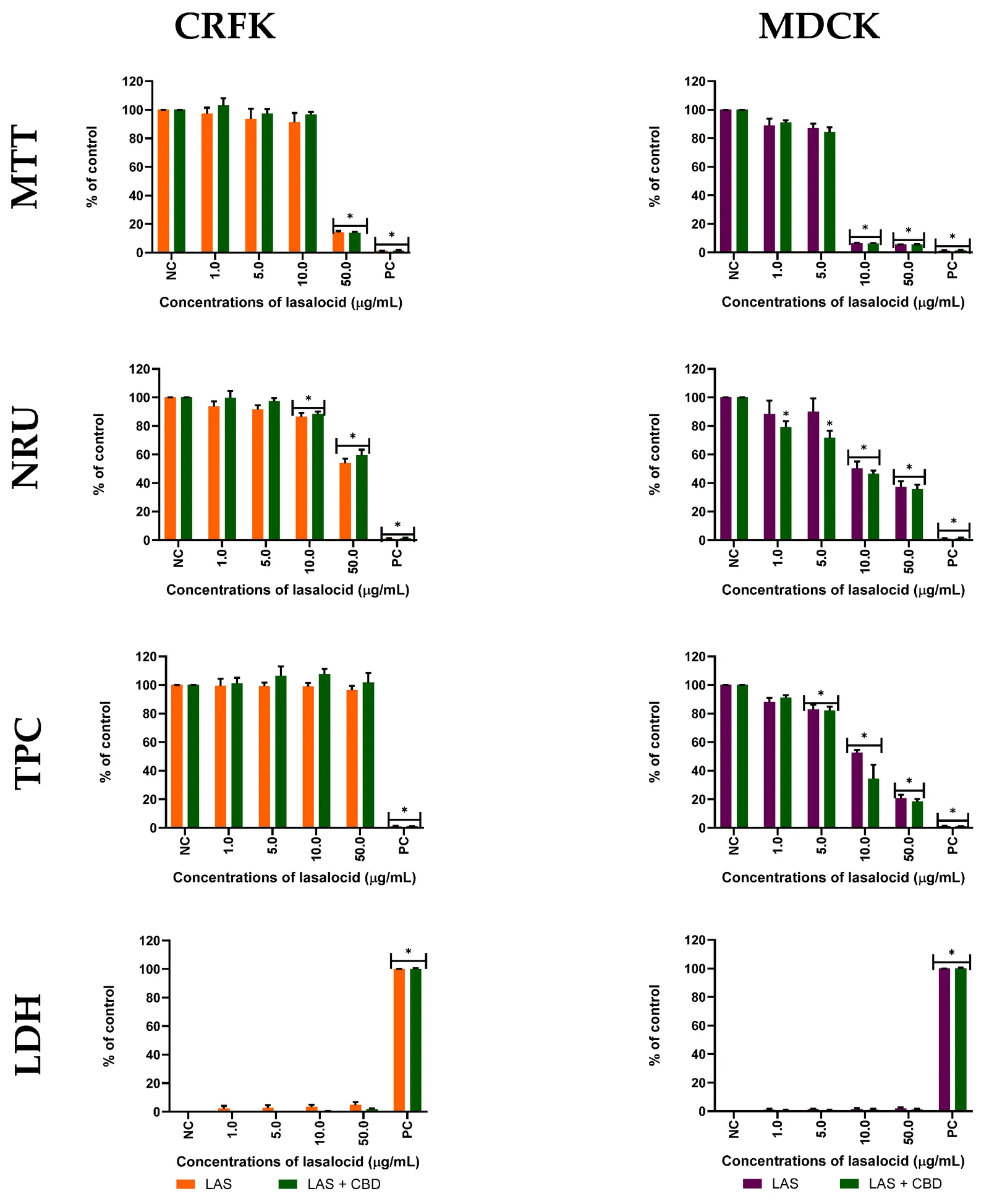
The inhibitory effect of lasalocid (LAS) and its interaction with cannabidiol (CBD) at concentrations of 1.56 µg/mL are shown in two kidney cell lines from cats (CRFK) and dogs (MDCK). The study was performed using four assessment tests: mitochondrial activity (MTT assay), lysosomal activity (NRU assay), proliferation (TPC assay), and cell membrane integrity (LDH assay). Data are presented as mean ± SD (standard deviation), *n* = 3. NC—negative control (0.1% DMSO in medium for LAS or 0.2% DMSO in medium for LAS + CBD); PC—positive control (1% Triton X-100 in medium). * *p* ≤ 0.05.

Lasalocid inhibited lysosomal and mitochondrial activity in CRFK cells, starting at concentrations of 10.0 and 50.0 µg/mL, respectively (*p* ≤ 0.05) (Figure 3). The presence of CBD increased the drug concentration required to inhibit the assessed endpoints (Figure 3). In CRFK cells, the lowest IC_50_ values for the drug and its mixture with CBD were observed only in the MTT assay (Table 2). An antagonistic interaction between LAS and CBD was observed in inhibiting lysosomal activity (NRU), cell proliferation (TPC), and disruption of cell membrane integrity (LDH). However, at high drug concentrations (50 µg/mL), an additive interaction was observed in the MTT assay (Figure 2).

**Figure 4:**
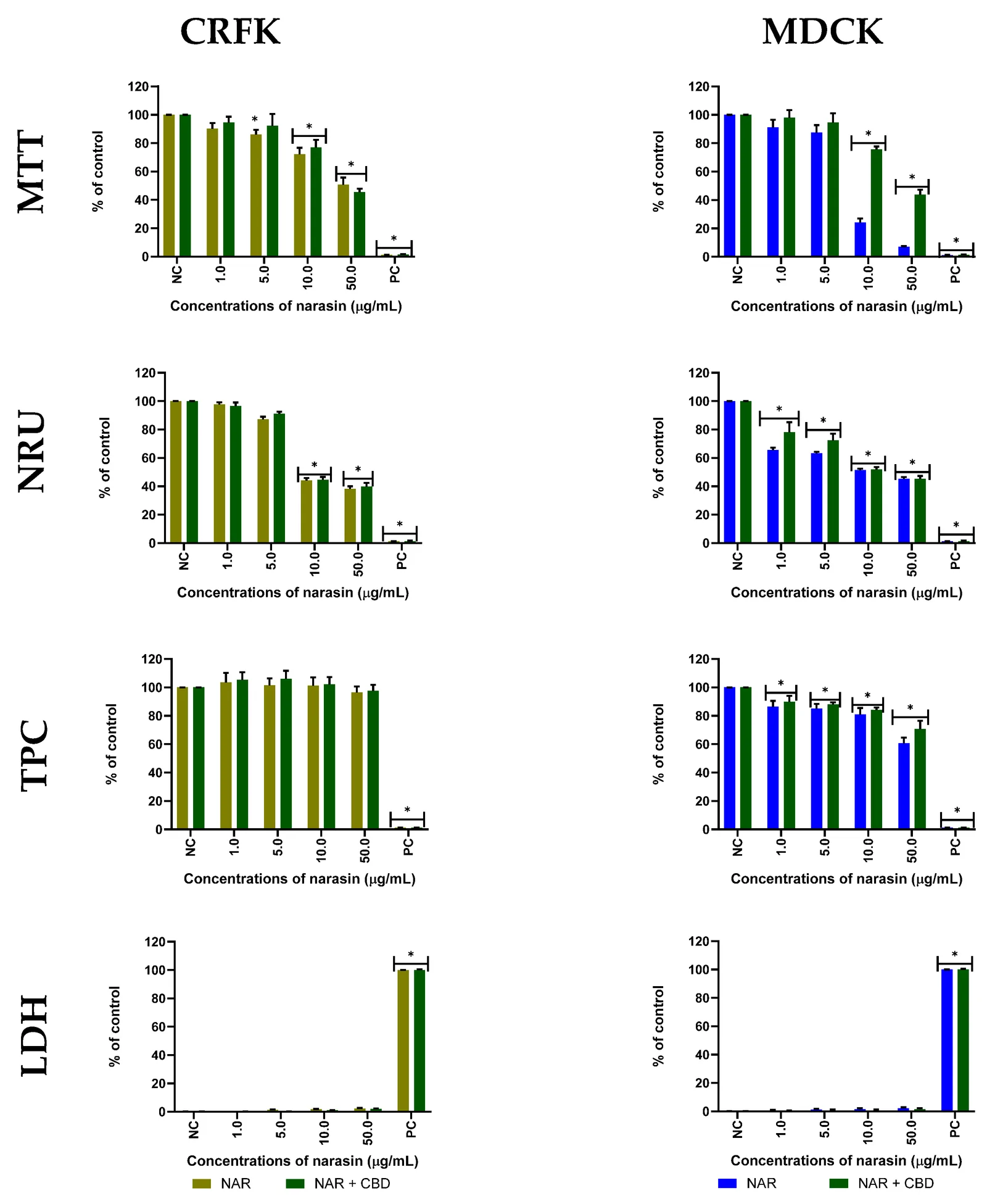
The inhibitory effect of monensin (MON) and its interaction with cannabidiol (CBD) at concentrations of 1.56 µg/mL are shown in two kidney cell lines from cats (CRFK) and dogs (MDCK). The study was performed using four assessment tests: mitochondrial activity (MTT assay), lysosomal activity (NRU assay), proliferation (TPC assay), and cell membrane integrity (LDH assay). Data are presented as mean ± SD (standard deviation), *n* = 3. NC—negative control (0.1% DMSO in medium for NAR or 0.2% DMSO in medium for NAR + CBD); PC—positive control (1% Triton X-100 in medium). * *p* ≤ 0.05.

The effect of lasalocid on MDCK cells differed from that observed in CRFK cells. In MDCK cells, the drug inhibited proliferative activity from a concentration of 5 µg/mL and mitochondrial and lysosomal activity from a concentration of 10 µg/mL. Co-treatment with CBD reduced lysosomal activity at the lowest concentration tested (1 µg/mL) (Figure 4). In the TPC assay, an almost sevenfold lower IC_50_ value was obtained for the mixture compared to the drug alone. Interaction analysis revealed a concentration-dependent effect (Figure 3, Table 3). Inhibition of mitochondrial activity (MTT) at the lowest concentration tested (1 µg/mL) resulted in an additive effect, while higher concentrations induced a synergistic effect. A similar pattern was observed for inhibition of proliferation (TPC): at the lowest concentration tested (1 µg/mL), an additive effect occurred, while higher concentrations induced a synergistic effect. Inhibition of lysosomal activity (NRU) and disruption of cell membrane integrity (LDH) resulted in a slight synergistic effect in MDCK cells (Figure 2).

### 2.4. The Effects of Monensin and Its Interaction with CBD in Feline Kidney (CRFK) and Dog (MDCK) Cells

Monensin inhibited mitochondrial and lysosomal activity in CRFK cells, starting at concentrations of 1.0 and 5.0 µg/mL, respectively (*p* ≤ 0.05) (Figure 4). The presence of CBD increased mitochondrial and proliferative activity and reduced the degree of membrane integrity impairment, whereas lysosomal activity showed a different pattern (see below). In CRFK cells, the lowest IC_50_ values for the drug and its mixture with CBD were observed only in the NRU assay (Table 2). An antagonistic interaction between MON and CBD was observed in inhibiting mitochondrial activity (MTT), cell proliferative activity (TPC), and disruption of cell membrane integrity (LDH). However, an additive interaction was observed in inhibiting lysosomal activity (NRU) (Figure 2).

The effects of monensin in MDCK cells showed both similarities and differences compared to CRFK cells. In MDCK cells, the drug inhibited lysosomal activity from a concentration of 1.0 µg/mL and mitochondrial activity from a concentration of 5.0 µg/mL—the reverse of the sensitivity order observed in CRFK cells (Figure 4). Co-treatment with CBD increased the activity of all tested cellular parameters. Similar to CRFK cells, the IC_50_ for MON and its mixture with CBD was calculated in the NRU assay; these values were almost three-fold higher than those calculated in CRFK cells (Table 2). As in CRFK cells, interaction analysis revealed an antagonistic effect in the inhibition of mitochondrial (MTT) and proliferative (TPC) activity. Unlike in CRFK cells, however, inhibition of lysosomal activity (NRU) at low concentrations (1.0 and 5.0 µg/mL) resulted in an antagonistic effect, while higher concentrations induced an additive effect. Also, unlike in CRFK cells—where this endpoint showed an antagonistic interaction—disruption of cell membrane integrity (LDH) in MDCK cells showed an additive effect independent of drug concentration (Figure 2).

### 2.5. The Effects of Narasin and Its Interaction with CBD in Feline Kidney (CRFK) and Dog (MDCK) Cells

**Figure 5:**
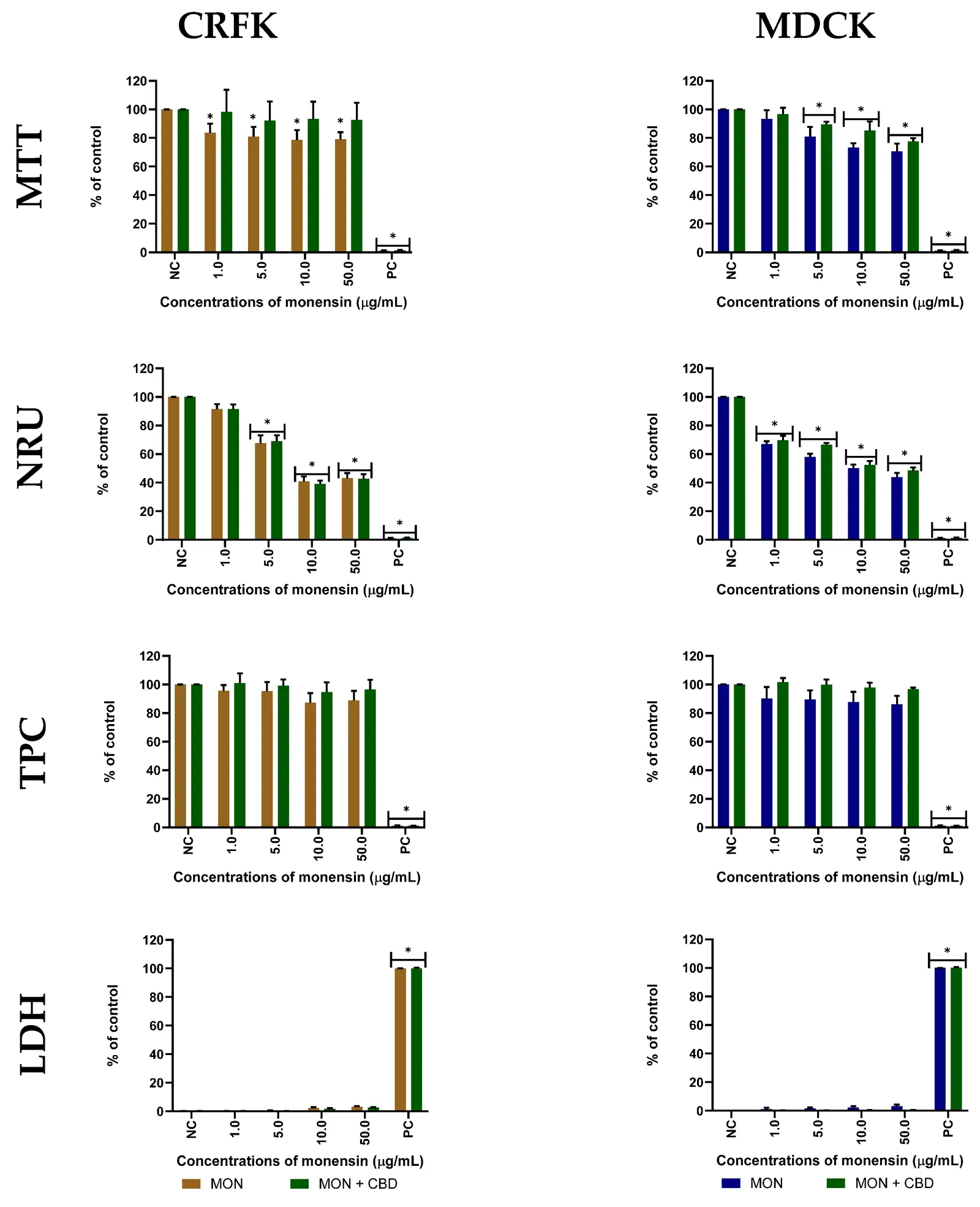
The inhibitory effect of narasin (NAR) and its interaction with cannabidiol (CBD) at concentrations of 1.56 µg/mL are shown in two kidney cell lines from cats (CRFK) and dogs (MDCK). The study was performed using four assessment tests: mitochondrial activity (MTT assay), lysosomal activity (NRU assay), proliferation (TPC assay), and cell membrane integrity (LDH assay). Data are presented as mean ± SD (standard deviation), *n* = 3. NC—negative control (0.1% DMSO in medium for MON or 0.2% DMSO in medium for MON + CBD); PC—positive control (1% Triton X-100 in medium). * *p* ≤ 0.05.

Narasin inhibited mitochondrial and lysosomal activity in CRFK cells starting from concentrations of 5.0 and 10.0 µg/mL, respectively (*p* ≤ 0.05) (Figure 5). The presence of CBD increased the mitochondrial activity of cells at low NAR concentrations, while at high drug concentrations (50.0 µg/mL), a decrease in this activity was observed. Regardless of the drug concentration, an increase in lysosomal and proliferative activity was also observed, and the degree of cell membrane integrity impairment was reduced. In CRFK cells, the lowest IC_50_ values were observed in the NRU assay; a finite IC_50_ was also obtained for the NAR + CBD mixture in the MTT assay, but not for NAR alone (Table 2). An antagonistic nature of the interaction between NAR and CBD in inhibiting mitochondrial activity (MTT) was observed, and a synergistic effect was observed only at high drug concentrations. The interaction was additive for lysosomal activity (NRU), borderline additive/antagonistic for cell proliferation (TPC), and antagonistic for cell membrane integrity (LDH) (Figure 2, Table 3).

In MDCK cells, narasin inhibited lysosomal and proliferative activity from a concentration of 1.0 µg/mL and mitochondrial activity from a concentration of 10.0 µg/mL. Co-treatment with CBD resulted in an increase in the activity of all tested cellular parameters (Figure 5). The IC_50_ value for narasin alone in the MTT assay was about seven times lower than for its mixture with CBD (Table 2). Interaction analysis revealed an antagonistic effect in inhibiting mitochondrial (MTT) and proliferative (TPC) activity. Similarly, inhibition of lysosomal activity (NRU) showed an antagonistic effect at low concentrations (1 and 5 µg/mL), but this became additive at higher concentrations. An additive effect, independent of drug concentration, was observed in the disruption of cell membrane integrity (LDH) in MDCK cells (Figure 2).

### 2.6. Summary of the CBD–Coccidiostat Interactions

The interaction classifications for all four coccidiostats across both cell lines and all four endpoints are collected in Table 3. Three patterns are apparent only when the results are considered together. In CRFK cells, the interaction was predominantly antagonistic or additive, with CBD increasing the concentration of each coccidiostat required to inhibit most endpoints, and synergy arising only at the highest concentrations (MTT for SAL and NAR; LDH for SAL). In MDCK cells, the outcome was compound-dependent rather than uniform. SAL and LAS were potentiated by CBD across most endpoints, whereas MON and NAR retained the antagonism seen in the feline line. Finally, in most combinations whose classification varied with concentration, the shift ran from antagonism or additivity at low concentration toward additivity or synergy at higher concentrations; the exceptions were SAL in the NRU assay in CRFK cells (antagonistic only at 5 µg/mL) and LAS in the NRU assay in MDCK cells (synergistic at 1–10 µg/mL and borderline at 50 µg/mL).

### 2.7. Molecular Docking Results

#### 2.7.1. Individual Ligand Screening

Each compound was first docked independently (Table 4). The four ionophores interacted with predicted affinities ranging from −8.24 to −7.79 kcal/mol. Cannabidiol interacted more weakly (−6.79 ± 0.16 kcal/mol). The reference ligands interacted within the same range as the ionophores: β-NADH (−8.93 ± 0.16), VBIT-4 (−8.14 ± 0.29), and SW016789 (−7.73 ± 0.04 kcal/mol). Standard deviations across three independently seeded runs ranged from ±0.04 to ±0.39 kcal/mol for single-ligand conditions (up to ±0.74 kcal/mol for multi-ligand assemblies), indicating adequate convergence of the search.

**Figure 6:**
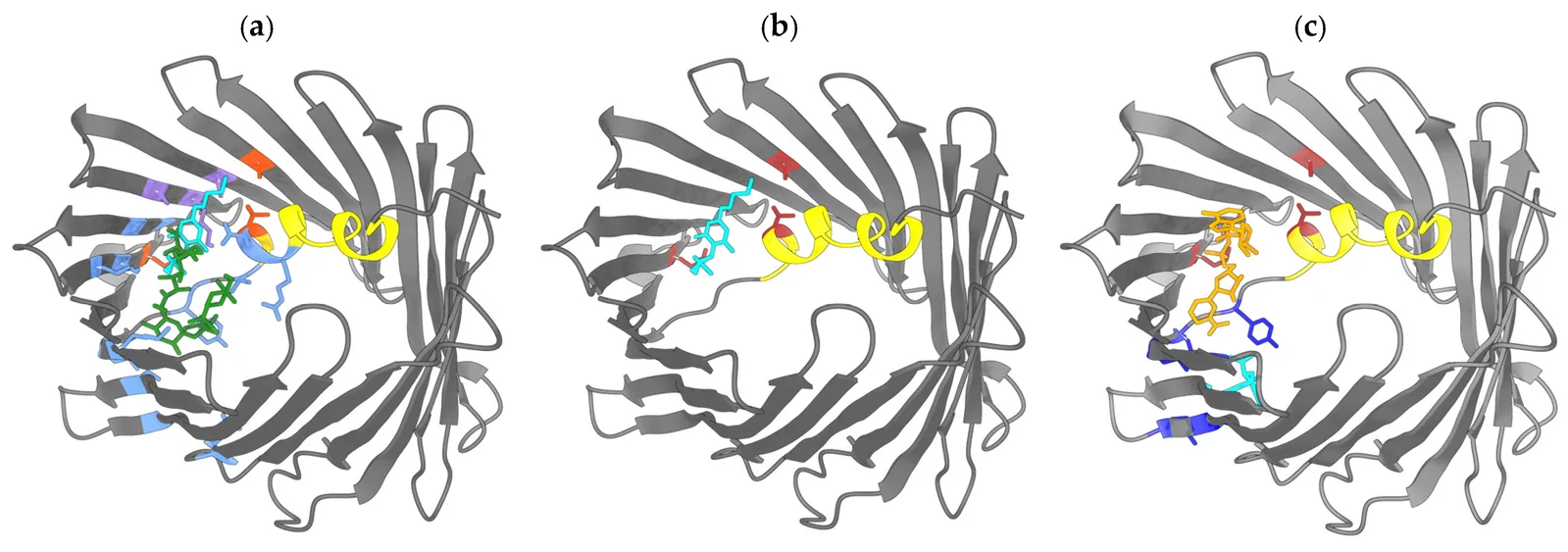
Predicted binding sites of cannabidiol and the ionophore coccidiostats on VDAC1. (**a**) The VDAC1 (grey) viewed down the pore axis, with the N-terminal α-helix (residues 5–19) in yellow. Interacting residues are shown in three categories: interacting with the ionophores only (blue; Arg15, Asp16, Thr19, Gly21, Tyr22, Leu26, and Lys28 in the N-terminal helix, linker, and β1; Ser46, Thr55, and Asn76 in β2–β4; and Gly244, Gly246, Thr258, and Ser260 in β17–β18); interacting with CBD only (orange-red; Val17, Ala205, and Leu262); and interacting with both (purple; Ala222, Ser234, and Lys236). The top-ranked poses of SAL (green) and CBD (cyan) are shown as sticks. (**b**) Top-ranked CBD pose (cyan) when docked alone, occupying the C-terminal region, with its interacting residues Val17, Ala205, and Leu262 (brown). (**c**) The same view of the CBD–β-NADH simultaneous docking: β-NADH (orange) occupies the C-terminal region while CBD (cyan) is displaced to a separate pocket formed by Tyr22, Phe24, Leu26, Lys53, and Thr55 (blue).

All four ionophores engaged in two regions of the barrel simultaneously (Table 4, Figure 6). Every compound had a hydrophobic interaction with Lys28 in β1, and three of the four formed hydrogen bonds within the N-terminal α-helix (residues 5–19) [28]. SAL and NAR with Arg15 and Thr19, and MON with Asp16. SAL additionally hydrogen-bonded Gly21 in the helix–β1 linker. LAS formed no hydrogen bonds in this region, approaching it only through hydrophobic contacts with Tyr22, Leu26, and Lys28. All four also interacted with the C-terminal strands β15–β18, three of them reaching Lys236 in β16 and Ser260 in β18. MON was the only individually docked compound to extend into β2–β4, hydrogen-bonding Ser46 and Asn76, and hydrophobic interaction with Thr55, and it formed more hydrogen bonds (five) than any other ionophore. Secondary-structure assignments throughout follow the HELIX and SHEET records of PDB 6TIQ [29].

CBD showed a distinct distribution. Five of its six interactions lie in the C-terminal half of the barrel—Ala205 (β14), Ala222 (β15), Ser234 and Lys236 (β16), and Leu262 (β18)—with a single N-terminal contact at Val17 and only one hydrogen bond to Ser234. Its interaction profile was dominated by hydrophobic contacts. Under identical docking conditions, the predicted CBD site (Ser234) overlapped that of the reference ligand β-NADH at Ser234, Lys236, and the β18 region and only partially overlapped the N-terminal helix region engaged by all four ionophores, with Val17 as its single N-terminal contact. The reference oligomerization inhibitor VBIT-4 contacted both regions (Tyr22, Gly23, Phe24, Leu26, Lys236, and Ser260), while the reference activator SW016789 contacted Lys28, Lys236, and Thr258.

#### 2.7.2. Simultaneous Molecular Co-Docking of CBD with Each Ionophore

Simultaneously docking CBD with each ionophore and with β-NADH (Table 4 and Table 5) yields binding affinity scores that are composite values for the whole assembly and include inter-ligand interaction terms; the scores were therefore interpreted against the sum of the corresponding individual scores and by ligand efficiency (Table 5). Based on this, every co-docked complex was predicted to bind less favorably than the sum of its constituent parts. CBD + SAL −10.23 ± 0.23 against a summed −14.58 kcal/mol (difference +4.35), CBD + LAS −10.98 ± 0.34 against −15.00 (+4.02), CBD + MON −10.75 ± 0.15 against −14.88 (+4.13), CBD + NAR −9.94 ± 0.61 against −15.03 (+5.09), and CBD+β-NADH −10.56 ± 0.23 against −15.72 kcal/mol (+5.16). Ligand efficiency fell in every case, from 0.147 to 0.195 kcal/mol per heavy atom for the individual ionophores to 0.129–0.169 kcal/mol per heavy atom for the assemblies. The predicted interaction between CBD and each co-ligand at this site is therefore one of mutual interference rather than cooperative binding. In each CBD–ionophore pair, the ionophore retained or strengthened its engagement of the N-terminal helix and β1—SAL, forming a salt bridge with Lys28 (2.98 Å), LAS with Lys28 (4.06 Å, including π-cation interaction with this residue with a bond length of 4.17 Å), MON with Arg15 (4.78 Å), and NAR with Lys28 (3.93 Å).

The CBD–β-NADH condition showed this asymmetry most clearly. β-NADH retained essentially the whole of its individual binding mode, hydrogen-bonding Ser234, Lys236, Leu245, Gly246, Thr248, Ser260, and Ala261, and forming salt bridges with Lys236 (5.29, 4.77 Å), while additionally engaging Asp16 and Tyr22. CBD, by contrast, was displaced out of the C-terminal region it occupied when docked alone (Ala205, Ala222, Ser234, Lys236, Leu262) into a distinct pocket formed by Tyr22, Phe24, Leu26, Lys53, and Thr55, hydrogen-bonding Thr55. The same relocation was observed in the CBD–LAS pair. Within this simulation set, therefore, when CBD and β-NADH are present together, β-NADH occupies the shared region, and CBD relocates, which is a more direct indication of competition between them than the static overlap of their separately docked sites.

#### 2.7.3. Five-Ligand Competition

To separate the contribution of CBD from that of ionophore crowding alone, the four ionophores were docked simultaneously both with and without CBD (Table 4 and Table 5). Without cannabidiol, the four-ligand assembly (196 heavy atoms, 63 active torsions) scored −11.49 ± 0.74 kcal/mol against a summed individual value of −32.33 kcal/mol. With CBD, the five-ligand assembly (219 heavy atoms, 71 active torsions) scored −12.53 ± 0.16 against −39.12 kcal/mol. Ligand efficiency was 0.059 and 0.057 kcal/mol per heavy atom, respectively. Adding CBD to the already-saturated four-ionophore assembly improved the composite score by only 1.04 kcal/mol, compared with the −6.79 kcal/mol it achieved when docked alone, approximately 15% of its individual binding energy, with no gain in ligand efficiency. The displacement of ligands from the primary pocket onto a peripheral surface of the barrel occurred in the absence of CBD. In the four-ionophore run, the ligands contacted Met1, Ala2, Val3, Thr19, Lys28, Arg93, and Lys119, essentially the same peripheral surface seen in the five-ligand run (Met1, Ala2, Tyr67, Ala92, Arg93, Leu95, Lys119). Displacement under saturating co-occupancy is therefore most likely a consequence of crowding among the ionophores themselves, not of cannabidiol.

## 3. Discussion

The presence of ionophore coccidiostats in cat and dog food can cause adverse effects and even death, as reported in the literature [5,6,7,8,9,10,11,12,13]. Among the clinical signs and post-mortem lesions described, renal damage has also been reported. The present study focused on evaluating the degree of damage to feline and canine renal cells and the protective effect of cannabidiol. The results indicate a differential nephrotoxic potential of salinomycin (SAL), lasalocid (LAS), monensin (MON), and narasin (NAR) in feline (CRFK) and canine (MDCK) renal epithelial cell lines. In addition, they reveal that the concurrent administration of cannabidiol (CBD) significantly modulates the evaluated cellular parameters, acting either as a cytoprotective agent or as a potentiator of nephrotoxic effects, depending on the coccidiostat and the cell model. Cannabidiol was not inert at the concentration used in the combination experiments: at 1.56 µg/mL, it inhibited lysosomal activity and total protein content in CRFK cells, whereas in MDCK cells, it slightly increased mitochondrial activity and total protein content. Its intrinsic activity is therefore probably species- and endpoint-dependent, which contributes to divergent combination outcomes rather than standing apart from them.

In the feline CRFK cell model, the tested coccidiostats exhibited variable cytotoxic potential, with a clear divergence in the sensitivity of individual cellular parameters. SAL strongly induced a concentration-dependent decrease in metabolic activity compared with the inhibition of lysosomal activity. Co-treatment with CBD significantly increased the SAL IC_50_ values in both the MTT and NRU assays in CRFK cells (Table 2), although combination index analysis indicated additive to synergistic interactions at the highest SAL concentrations (Table 3). This partial protective action suggests that CBD may mitigate SAL-induced cell injury in the feline renal epithelium at lower concentrations, potentially through well-established anti-inflammatory and antioxidative mechanisms that stabilize mitochondrial kinetics. Conversely, MON and NAR displayed a completely opposite cytotoxicity profile in CRFK cells, in which lysosomes represented the primary target of injury. Both compounds exhibited high toxic potency in the NRU assay, with very low IC_50_ values (6.7 ± 0.7 µg/mL for MON and 5.7 ± 0.2 µg/mL for NAR), whereas their mitochondrial toxicity remained largely undetectable within the tested concentration range. Concurrent administration of CBD showed an additive interaction with respect to the lysosomal activity of MON and NAR. In contrast, the presence of CBD was found to sensitize feline cells to NAR-induced mitochondrial damage. LAS proved to be the least toxic coccidiostat in the feline model, with CBD exerting an antagonistic (protective) interaction with respect to lysosomal activity and proliferative capacity.

The sensitivity of feline renal cells to the direct effects of coccidiostats provides important mechanistic context for the in vivo data. In the reported case of cat poisoning caused by SAL-contaminated feed, post-mortem analysis confirmed high concentrations of the coccidiostat in the liver and kidneys [6,9]. Although the dominant clinical signs reported in that study strongly indicated peripheral polyneuropathy rather than overt, morphologically evident renal failure, our in vitro data clearly indicate that feline renal epithelial cells possess an inherent metabolic susceptibility to these compounds. This discrepancy suggests that subclinical, functional cellular disturbances within the kidney may occur independently of, or prior to, the development of overt histopathological changes in the whole organ. The cytotoxic response of canine MDCK cells differed fundamentally from that observed in the feline model, particularly with regard to the interaction of CBD with SAL, which showed relatively low cytotoxicity within the tested concentration range in MDCK cells. However, the presence of CBD triggered a profound, synergistic potentiation of toxicity, reducing mitochondrial, lysosomal, and proliferative cell activity. This dramatic shift indicates that, although canine renal cells can effectively tolerate SAL alone in vitro, the addition of CBD completely overwhelms their homeostatic capacity. This destructive synergy is likely related to the underlying mechanism of ionophore action: SAL severely disrupts transmembrane potassium (K^+^) transport, forcing a secondary, toxic influx of intracellular calcium (Ca^2+^), which causes mitochondrial swelling, collapse of the membrane potential, and an uncontrolled increase in reactive oxygen species (ROS) [30]. Given that CBD also acts on mitochondria by regulating intracellular Ca^2+^ dynamics, this interaction may be related to lethal cellular calcium overload [14,15]. A similar trend of strong cytotoxicity potentiation by CBD was observed for LAS in MDCK cells. LAS induced marked inhibition of both mitochondrial and lysosomal cell activity, whereas the presence of CBD elicited a pronounced antiproliferative effect. Isobolographic analysis confirmed that CBD combined with higher concentrations of LAS produced clear synergistic effects across all cellular parameters. This high sensitivity of canine renal cells to LAS strongly corresponds with the clinical signs of in vivo canine poisoning. Segev et al. [31] documented the accidental poisoning of dogs that had consumed feed contaminated with LAS (~200 mg/kg), which resulted in rapid flaccid paralysis and hyporeflexia, leading to high mortality and establishing a remarkably low oral LD_50_ of only 10–15 mg/kg in dogs. Furthermore, MON and NAR exhibited distinct, separate profiles in the canine model. MON inhibited lysosomal activity almost three-fold less potently than in feline cells, indicating a lower sensitivity of MDCK cells to direct MON-induced lysosomal lysis. NAR strongly inhibited MDCK mitochondrial activity, whereas the presence of CBD exerted a protective effect on this parameter. MON poisoning in dogs is well documented by Hazlett et al. [5], who described severe, bilateral renal medullary necrosis in puppies following the ingestion of MON-contaminated dog food. In animals that survived the initial toxic syndrome, chronic renal manifestations included extensive interstitial cortical fibrosis, mineralization of tubules and glomeruli, and extensive loss of functional nephrons.

Across every condition tested, no LDH IC_50_ was reached, indicating that these interactions occur without loss of plasma membrane integrity and therefore at an intracellular level. Molecular docking was used to explore a possible molecular basis. Both compound classes were accommodated on VDAC1, but only in partially overlapping regions (Table 4, Figure 6). The ionophores engaged the N-terminal α-helix and β1, in addition to the C-terminal strands, whereas CBD was confined to the C-terminal half. Shared occupancy at three residues, alongside exclusive occupancy elsewhere, would be expected to yield a mixed competitive and non-competitive interaction rather than simple competition. Every co-docked complex also scored lower than the sum of its constituents, suggesting that CBD and the ionophores hinder rather than assist one another at this site. This is consistent with the antagonism that predominated in CRFK cells, but not with the potentiation of SAL and LAS in MDCK cells, for which the calcium-overload mechanism outlined above remains the more likely explanation. CBD is chemically different from the other compounds it was tested against. At 314.5 g/mol, it is less than half the mass of the ionophores, carries a fraction of their polar surface area, and has neither a polyether cage nor a carboxylate group (Table 5). It therefore cannot chelate a cation or act as an ionophore, and its combination of high lipophilicity with very low polar surface area is instead the profile of a hydrophobically driven, membrane-partitioning binder, as shown by the molecular docking result, which shows a single hydrogen bond and five hydrophobic contacts.

The single canine–feline substitution identified in the alignment offers one candidate for this species dependence. The two residues are similar in size and shape, but they are not functionally equivalent: the feline channel loses a hydroxyl group annotated as a phosphorylation site in the human protein (UniProtKB entry P21796) [32] and gains a thiol of the kind that, in VDAC isoforms, is a documented target of oxidation by reactive oxygen species [33,34]. This matters because phosphorylation at other positions is known to regulate VDAC1 conductance and gating [35,36].

A regulatory difference of this kind, on the same helical face that the docked ligands engage, could plausibly change how the channel responds when both compounds are bound, and its effect would be greatest under the oxidative conditions these ionophores create. We present this as a hypothesis generated by the alignment rather than a mechanism demonstrated here: the UniProtKB entry assigns no kinase to this site [32], and we are not aware of any functional characterization of the feline orthologue.

The application of a multi-endpoint assay system (MTT, NRU, TPC, and LDH) in species-specific epithelial cell lines provides an important discriminatory capacity that is frequently lacking in conventional in vivo screening. Long-term (90-day) exposure of Beagle dogs to certain novel coccidiostats (e.g., ethanamizuril) [37] has been shown to induce distinct histopathological changes in the kidneys without causing significant changes in conventional serum biomarkers of renal function, such as blood urea nitrogen or creatinine. This demonstrates that coccidiostat-induced renal damage can progress to hazardous tissue-level pathology long before routine clinical blood chemistry signals organ failure. In vitro assays can therefore provide a highly sensitive screen for the tested compounds at the early stages of tissue or organ damage before systemic signs occur. It should be noted that the differences observed between CRFK and MDCK cells highlight that the toxicity of ionophore coccidiostats and its modulation by phytocannabinoids cannot be generalized across companion animal species. As emphasized by Ekinci et al. [30], despite a shared general mechanism of action involving transmembrane ion transport, the specific toxicological outcomes and resulting clinical phenotypes differ substantially between animal species owing to differences in metabolic rate, cell membrane lipid composition, and receptor sensitivity. Although CBD shows promising cytoprotective activity against the toxicity of certain veterinary drugs in human models (such as HEK-293) [25,26,27] or in feline cells, its strong synergistic potentiation of SAL and LAS toxicity in canine cells raises urgent safety concerns.

This study has some limitations that should be considered when interpreting the results. The experiments were conducted under in vitro conditions using selected canine and feline cell lines, and therefore, the findings may not fully reflect the complex responses observed in vivo. In addition, the study focused on selected concentrations and experimental endpoints, which may not encompass the full range of possible biological effects of coccidiostats and CBD. The stability and metabolic fate of CBD under the assay conditions were not determined. CBD is sensitive to oxidation and degrades appreciably in aqueous media at physiological pH and temperature, with approximately 10% lost within 24 h at pH 7.4 and 37 °C [38], and it is extensively protein-bound, with an unbound fraction of about 5% at low concentrations in plasma [39]. The nominal concentration of 1.56 µg/mL, therefore, represents an upper bound on the free concentration available during the 72 h exposure. Both cell lines also differ substantially from primary renal tissue. MDCK and comparable lines lack the SLC22 and SLCO organic anion transporters and show much lower brush-border enzyme activity [40], while CRFK are fibroblast-like rather than tubular epithelial [41]. Uptake and biotransformation of the test compounds in this study, therefore, do not need to reflect those in vivo, and whether the ionophores influence CBD stability or metabolite formation was not determined. Further studies using complementary experimental models and approaches, particularly those investigating mechanisms related to oxidative stress, mitochondrial function, ionic homeostasis, cellular uptake, or the metabolism of CBD/ionophores, could help elucidate the biological significance of the present findings and extend them further. Three experiments would specifically test the VDAC1 hypothesis. Target validation by VDAC1 knockdown or co-treatment with the oligomerization inhibitor VBIT-4, extracellular flux analysis to establish whether CRFK and MDCK cells differ in their reliance on oxidative phosphorylation, which would determine whether reduced channel conductance is protective or injurious in each, and site-directed substitution at position 13 combined with bilayer electrophysiology.

Several limitations of the computational component should also be noted. Molecular docking was performed on a membrane-free receptor, whereas VDAC1 is an outer-membrane beta-barrel [42,43]; given CBD’s high lipophilicity (cLogP 5.85), its physiologically relevant interaction may occur at the lipid-protein interface rather than within the pore lumen. A single rigid-receptor docking pose cannot establish allosteric coupling between the two sites, which would require molecular dynamics of the ternary complex (outside the scope of this present study). Scores from simultaneous multi-ligand docking are composite values that include inter-ligand interaction terms and are not directly comparable to single-ligand scores.

## 4. Materials and Methods

### 4.1. Materials

Analytical standard of cannabidiol (CAS 13956-29-1; molecular weight 314.5 g/mol; Cat. No. 85705, PhytoLab GmbH & Co. KG (Vestenbergsgreuth, Germany)) and coccidiostats such as lasalocid sodium salt (LAS; CAS 25999-31-9; molecular weight 590.79 g/mol; >90%), monensin sodium salt hydrate (MON; CAS 22373-78-0; molecular weight 692.85 g/mol; 90–95%), narasin sodium salt (NAR; CAS 55134-13-9; molecular weight 765.03 g/mol; 98%) and salinomycin monosodium salt (CAS 55721-31-8; molecular weight 772.98 g/mol; Cat. No. 46729) were purchased from Sigma-Aldrich, Poznan, Poland. Reagents needed for cell culture and testing, such as: Triton X-100, trypan blue, DMSO, FBS, neutral red (NR), Coomassie Brilliant Blue R-250, MTT reagent (3-(4,5-dimethylthiazol-2-yl)-2,5-diphenyltetrazolium bromide), trypsin–EDTA, L-glutamine, antibiotic solution (10,000 U/mL penicillin, 10 mg/mL streptomycin), and Cytotoxicity Detection Kit (LDH), were purchased from Sigma-Aldrich, Poznan, Poland. All other reagents were purchased from commercial suppliers and were of the highest available purity.

### 4.2. Cell Line Cultures

The study used commercial cell lines purchased from the American Type Culture Collection (ATCC, Manassas, VA, USA): MDCK, Madin-Darby canine kidney—epithelial cells derived from canine kidney (ATCC CCL-34); and CRFK, Crandell-Rees feline kidney (ATCC CCL-94). Cells were cultured in MEM (Sigma-Aldrich Poznan, Poland) with 10% FBS, 1% L-glutamine, and 1% antibiotic at 37 °C, 5% CO_2_ in 75 cm^2^ NUNC flasks. Medium was changed every 2–3 days; cells were passaged at 70–80% confluence using 0.25% trypsin–0.02% EDTA. Single-cell suspensions (1 × 10^5^ cells/mL) were seeded into 96-well plates (100 µL/well) and incubated for 24 h before exposure to the study drugs or their mixtures with CBD.

### 4.3. Exposure of Cells to Substances

Stock solutions of LAS, MON, NAR, SAL, and CBD were prepared in DMSO. Subsequently, working solutions were obtained by dilution in antibiotic-free culture medium supplemented with 1% FBS. The final concentrations used in the experiments were 1, 5, 10, and 50 µg/mL for the coccidiostats and 1.56 µg/mL for CBD. The concentrations selected for the present study were based on previously published data [25,44] and preliminary experiments. The final DMSO concentration in the culture medium was 0.1% for individual compounds and 0.2% for coccidiostat–CBD combinations. Cells exposed to 0.1% or 0.2% DMSO served as negative controls (NC), while cells treated with 1% Triton X-100 were used as the positive control (PC). The NC was matched to the solvent content of each treatment: 0.1% DMSO for cells exposed to a single compound (each coccidiostat or CBD alone) and 0.2% DMSO for cells exposed to coccidiostat–CBD combinations. The PC was identical in all experiments. Thus, the same NC and PC conditions were applied in all four assays (MTT, NRU, TPC, and LDH) and in both cell lines. In the MTT, NRU, and TPC assays, the results were expressed as a percentage of the corresponding NC (100%), whereas in the LDH assay, the PC (complete cell lysis) represented the maximum LDH release (100% cytotoxicity). Cell viability and cytotoxicity were evaluated after 72 h of exposure to the tested compounds, either individually or in combination with CBD. The culture medium was not replaced during the exposure period. Cell viability and cytotoxicity were subsequently determined using four assays, as described below.

### 4.4. MTT Assay

The MTT assay was used to evaluate mitochondrial metabolic activity. This method is based on the reduction of the tetrazolium salt MTT by metabolically active cells, primarily through mitochondrial NAD(P)H-dependent oxidoreductases, resulting in the formation of insoluble purple formazan crystals. The amount of formazan produced is proportional to the number of viable, metabolically active cells [45]. Following the 72 h exposure period, 10 µL of MTT solution (5 mg/mL in PBS) was added to each well, and the plates were incubated for 3 h. The MTT-containing medium was then removed, and the resulting formazan crystals were solubilized in 100 µL of DMSO. Plates were shaken for 15 min at room temperature to ensure complete dissolution of the crystals. Absorbance was measured at 570 nm using a Synergy 2 microplate reader (BioTek^®^ Instruments, Inc., Winooski, VT, USA), with blank correction. Cell metabolic activity was expressed as a percentage of the corresponding negative control [25].

### 4.5. Neutral Red Uptake Assay

Lysosomal activity and cell viability were assessed using the Neutral Red Uptake (NRU) assay. Neutral red is incorporated by viable cells and accumulates within lysosomes, whereas cells with impaired membrane integrity or lysosomal function show reduced dye accumulation [46]. At the end of the exposure period, the medium containing the tested compounds was aspirated, and the cells were gently washed with PBS. Subsequently, 100 µL of neutral red solution (50 µg/mL) was added to each well, and the cells were incubated for 3 h. The dye solution was then removed, and the wells were washed with PBS. Intracellular neutral red was extracted using a mixture of acetic acid, ethanol, and water (1:50:49, *v*/*v*/*v*). After shaking the plates for 10 min, absorbance was determined at 540 nm using a Synergy 2 microplate reader (BioTek^®^ Instruments, Inc., Winooski, VT, USA), with blank correction. Results were calculated relative to the negative control and expressed as a percentage [25].

### 4.6. Total Protein Content Assay

The Total Protein Content (TPC) assay was performed to estimate cell biomass based on the amount of cellular protein remaining after treatment [47]. Following exposure to the tested compounds, the culture medium was removed, and 100 µL of Coomassie Brilliant Blue R-250 solution was added to each well. The plates were shaken for 10 min to allow protein staining. The staining solution was subsequently discarded, and the cells were washed twice with 150 µL of acetic acid/ethanol/water solution (5:10:85, *v*/*v*/*v*). Next, 100 µL of 1 M potassium acetate was added to each well to release the protein-bound dye, and the plates were shaken for an additional 10 min. Absorbance was recorded at 595 nm using a Synergy 2 microplate reader (BioTek^®^ Instruments, Inc., Winooski, VT, USA), after subtraction of the blank value. Total cellular protein content was expressed as a percentage of the negative control [25].

### 4.7. Lactate Dehydrogenase Assay

Cell membrane integrity was assessed by measuring the extracellular release of lactate dehydrogenase (LDH), an intracellular enzyme released into the culture medium following damage to the cellular membrane [48]. LDH activity was determined using the commercially available Cytotoxicity Detection Kit (LDH; Roche Diagnostics, Mannheim, Germany) according to the manufacturer’s instructions. After the exposure period, 100 µL of cell-free culture medium from each well was transferred to a transparent flat-bottom 96-well plate. An equal volume (100 µL) of the reaction mixture was then added. The plate was incubated for 30 min at room temperature in the dark, after which the enzymatic reaction was terminated by adding 50 µL of 1 M HCl to each well. Absorbance was measured at 492 nm using a Synergy 2 Multi-Mode Microplate Reader (BioTek^®^ Instruments, Inc., Winooski, VT, USA), with a blank used as the reference [25]. LDH release was expressed as a percentage of the maximum release obtained in the PC (1% Triton X-100), which was taken as 100% cytotoxicity.

### 4.8. Assessment of Interaction Between Coccidiostats and Cannabidiol

In order to determine the types of interaction that occur when cells are exposed to combinations of study coccidiostats and CBD, a method established by Chou and Talalay [49] was chosen in this study. The dose-effect relationships of the individual and combined study substances were biometrically modeled using the Median-Effect Equation of the Mass Action Law as described [49].

fa/fu = (D/Dm)^m^ (1)

D—dose of the drug or CBD; fa—fraction affected by D; fu—fraction unaffected (i.e., fu = 1 − fa); Dm—median-effect dose (e.g., IC_50_); m—coefficient signifying the shape of the dose-effect relationship (m = 1, m > 1, and m < 1 indicate hyperbolic, sigmoidal, and flat sigmoidal dose-effect curves, respectively).

In this isobolographic analysis, the combination index (CI) is a quantitative parameter used to evaluate the type of interactions and the corresponding influential level among multiple compounds. For all combinations, CI values were generated over a range of fractions of cell viability affected (Fa) from 0.05 to 0.95 (5–95% toxicity). CI = 0.9–1.1, CI < 0.9, and CI > 1.1 indicate additive, synergistic, and antagonistic effects, respectively.

### 4.9. Molecular Docking Methodology and Target Justification

#### 4.9.1. Target Selection, Homology, and Oxidative Stress Justification

To investigate plausible mechanisms underlying the compound-specific toxicities and cytoprotective interactions of cannabidiol (CBD) and polyether ionophores, a structural approach was employed through molecular docking simulations.

Because experimentally resolved canine and feline VDAC1 structures are unavailable, the ligand-free refined solution nuclear magnetic resonance (NMR) structure of human VDAC1 (PDB ID: 6TIQ, model 1) was used as the structural receptor. Sequence comparison (EMBL-EBI Clustal Omega Multiple Sequence Alignment Percent Identity Matrix from Clustal 2.1 [50]) showed 99.65% identity (with conservative substitution at position 129 from CLUSTAL O (1.2.4) multiple sequence alignment) between human and canine VDAC1 and 99.29% identity (with differences at positions 13 and 129) between human and feline VDAC1 across the 283-residue canonical protein sequence. Canine and feline VDAC1 are therefore identical except at residue 13, which is serine in the dog, as in humans, and cysteine in the cat. Residue 13 lies within the N-terminal α-helix, resolved as three segments spanning residues 3–6, 7–9, and 12–19, and is adjacent to Lys12, an annotated ATP-binding residue, and is itself an annotated phosphorylation site [32,51,52] with implications addressed in the Discussion. Thus, 6TIQ provides a highly conserved structural framework for evaluating ligand interactions with this functionally relevant VDAC1 region in canine and feline models.

The databases, accession numbers, and structural data used in the molecular docking analysis were as follows. The VDAC1 amino acid sequences were retrieved from the NCBI Protein database [53] for human (accession number NP_003365.1) and cat (accession number XP_023110928.1), and from the UniProt Knowledgebase [32] for dog (UniProtKB A0A8C0RGS2; UniParc UPI0000000D72). Functional annotations of human VDAC1 (ATP-binding residues and phosphorylation sites) were taken from UniProtKB entry P21796 (VDAC1_HUMAN) [32]. The atomic coordinates of the receptor were obtained from the RCSB Protein Data Bank [54] under PDB ID 6TIQ, the refined solution NMR structure of human VDAC1 in detergent micelles determined in the absence of ligands [29]. This structure comprises the 19-stranded transmembrane β-barrel and the N-terminal α-helix located inside the pore; model 1 of the deposited NMR ensemble was used for docking. Canonical SMILES of CBD, the four ionophores (SAL, LAS, MON, and NAR), and the reference ligands (β-NADH, VBIT-4, and SW016789) were obtained from the PubChem Compound database [55].

VDAC1 functions as the critical outer mitochondrial membrane gatekeeper, regulating the flux of respiratory substrates, cations, and metabolites, and serving as a central execution hub for mitochondria-mediated apoptosis [56,57,58]. Beyond its role in metabolic flux, VDAC1 is critically involved in oxidative stress and in the control of reactive oxygen species (ROS) pathways [59,60]. It acts as the primary conduit for the release of superoxide anions from the mitochondrial intermembrane space into the cytosol [57]. Dysregulation or sustained closure of VDAC1 can lead to the accumulation of mitochondrial ROS, lipid peroxidation, and downstream oxidative damage, key hallmarks of cellular injury [60]. Exploring ligand binding to VDAC1 through this structural approach is therefore directly relevant to decoding both metabolic disruptions and ROS-mediated cytotoxicity.

#### 4.9.2. Receptor and Ligand Preparation Protocols

Initial macromolecular cleanup, removal of water molecules, and clean isolation of the target protein receptor chains were executed using Molegro Molecular Viewer [61,62]. Following isolation, structural preparation of both the receptor and ligand input files was performed using AutoDock version 4.2.6 [63], mirroring the validated structural modeling workflows described by Ejiohuo et al. [64,65].

For receptor preparation, the 6TIQ structure was inspected for missing atoms and repaired if needed. Polar hydrogens were added, and Kollman charges were assigned. The prepared receptor was saved in PDBQT format. Canonical SMILES for CBD, polyether ionophores, and reference controls were obtained from PubChem [55]. Three-dimensional ligand structures were generated using Corina Classic 3D [66,67] and saved in PDB format. Ligands were then prepared in AutoDock v4.2.6 by defining the root and torsional degrees of freedom before export in PDBQT format. Active torsions per ligand (TORSDOF record) were CBD 8, SAL 16, LAS 17, MON 14, NAR 16, β-NADH 18, VBIT-4 9, and SW016789 6.

#### 4.9.3. Computational Simulation Protocol and Target Site Definition

All in silico docking simulations were conducted using AutoDock Vina (v1.2.7) [68,69] at a default exhaustiveness of 8. To ensure an unbiased yet highly targeted search, the docking grid box was centered on the highest-ranking ligand-binding pocket predicted by the PrankWeb server [70]. The grid search box was set to X = −4.9674, Y = −29.4229, and Z = −23.9163, with dimensions 28 Å × 28 Å × 28 Å.

Four analyses were performed: (i) each compound docked individually; (ii) cannabidiol co-docked simultaneously with each ionophore and with β-NADH; (iii) the four ionophores co-docked with and without cannabidiol; and (iv) β-NADH (an endogenous VDAC1 modulator [56,71]), VBIT-4 (a VDAC1 oligomerization inhibitor [72,73]), and SW016789 (a VDAC1-targeting activator [74]) docked individually as reference ligands with reported VDAC1 activity.

All complexes and visualizations were created using UCSF ChimeraX v1.11 [75]. Protein-Ligand Interaction Profiler (PLIP) [76] was used to identify interactions in the complex. To account for stochastic variation in the global optimization procedure and assess reproducibility of the predicted binding configurations, each individual and simultaneous multi-ligand docking condition was performed in triplicate using independently assigned random seeds. Binding energies were extracted from the top-ranked poses and summarized as mean ± sample standard deviation (SD) across the three independent runs.

Affinities from multi-ligand runs are composite values for the whole assembly and include inter-ligand interaction terms [77], so they are not directly comparable with single-ligand scores. Multi-ligand conditions were therefore assessed against the sum of the corresponding individual scores and by ligand efficiency, calculated as −ΔG divided by the number of non-hydrogen atoms to permit comparison between ligands of differing size. Because Vina affinities are empirical scores rather than measured binding free energies and already incorporate a normalization for the number of rotatable bonds [68], ligand efficiency is used here as a relative descriptor applied consistently across conditions rather than as a thermodynamic quantity. Physicochemical descriptors were calculated from the canonical SMILES using RDKit version 2026.03.1 [78]: molecular weight, Crippen logP, topological polar surface area, Lipinski hydrogen-bond donor and acceptor counts, rotatable-bond count, ring count, and the number of non-hydrogen (heavy) atoms, the last of these being the denominator used for ligand efficiency. Two distinct flexibility counts are reported and should not be conflated. Rotatable bonds were computed by RDKit, whose definition excludes bonds to terminal atoms [78] and therefore does not count hydroxyl rotations. Active torsions were read directly from the TORSDOF record of the prepared ligand PDBQT files and are the values AutoDock Vina uses during the search. The two differ by the number of rotatable polar hydrogens in each molecule—for cannabidiol, 6 rotatable bonds against 8 active torsions, the difference being its two hydroxyl groups.

### 4.10. Statistical Analysis

The study was performed in three independent experiments. The obtained results are presented as mean values ± SD (standard deviation). The assessment of the cytotoxicity data used a one-way analysis of variance (ANOVA) followed by Dunnett’s post hoc test, which was applied to determine significance relative to the negative control. The cytotoxicity concentration (IC_50_) necessary for a 50% inhibition of cell viability by the drug or its mixtures with CBD was calculated using GraphPad Prism 11.0. The obtained results are presented as mean values ± SEM (standard error of the mean). Statistical comparisons among IC_50_ values were performed with the analysis of variance (ANOVA) followed by the Tukey test, and differences were considered statistically significant at *p* ≤ 0.05.

## 5. Conclusions

The nephrotoxic potential of salinomycin, lasalocid, monensin, and narasin differed between feline (CRFK) and canine (MDCK) kidney cell lines. Cannabidiol modulated these effects in a divergent manner, exerting a protective action against most compounds in feline cells, whereas in canine cells it strongly potentiated the toxicity of salinomycin and lasalocid. Molecular docking supported VDAC1 as a structurally plausible shared target, and a single amino acid difference between the canine and feline channels, at an annotated phosphorylation site within the N-terminal helix, offers a specific and testable hypothesis for this species dependence. These findings indicate that subclinical damage to renal cells may precede the histopathological and biochemical changes observed in vivo, supporting the value of the in vitro model applied in this study as a sensitive screening tool.

## Figures and Tables

**Table 1:** Effect of cannabidiol alone on cellular endpoints in feline (CRFK) and canine (MDCK) kidney cells after 72 h exposure. Values are expressed as a percentage of the negative control, except LDH, which is expressed as a percentage of cytotoxicity. Data are presented as mean ± SD (*n* = 3).

Cell Line	Conc. (µg/mL)	MTT	NRU	TPC	LDH
CRFK	NC	100 ± 0.12	99.9 ± 0.10	101 ± 0.09	0.2 ± 0.03
	1.56	103.4 ± 1.80	67.5 ± 4.38 ****	89.5 ± 3.66 ***	1.4 ± 0.83
	3.13	102.8 ± 2.16	61.1 ± 2.06 ****	84.8 ± 2.27 ****	1.9 ± 0.95 *
	6.25	98.9 ± 2.57	57.3 ± 3.67 ****	68.9 ± 3.53 ****	3.0 ± 0.47 ***
	12.5	86.3 ± 0.91 ****	29.4 ± 3.23 ****	66.6 ± 2.10 ****	3.1 ± 0.39 ***
	PC	0.2 ± 0.02 ****	0.1 ± 0.02 ****	1.2 ± 0.25 ****	100 ± 0.12 ****
MDCK	NC	101 ± 0.01	101 ± 0.02	101 ± 0.02	0.1 ± 0.01
	1.56	117.5 ± 4.62 ***	107.7 ± 3.60	107.7 ± 4.51 *	1.2 ± 0.77
	3.13	111.4 ± 3.61 **	107.5 ± 4.81	100.1 ± 2.88	1.2 ± 0.65
	6.25	103.2 ± 2.91	103.7 ± 3.26	99.8 ± 2.25	3.8 ± 2.42
	12.5	96.9 ± 4.71	80.3 ± 3.13 ****	91.7 ± 2.69 **	7.0 ± 3.14 **
	PC	0.5 ± 0.01 ****	1.3 ± 0.02 ****	1.2 ± 0.02 ****	100 ± 0.01 ****

NC—negative control (0.1% DMSO in medium); PC—positive control (1% Triton X-100 in medium). * *p* ≤ 0.05, ** *p* ≤ 0.01, *** *p* ≤ 0.001, **** *p* ≤ 0.0001 versus the negative control (one-way ANOVA with Dunnett’s post hoc test).

**Table 2:** IC_50_ values (µg/mL) calculated in CRFK and MDCK cell cultures using the MTT, NRU, TPC, and LDH assays after 72 h exposure to salinomycin (SAL), lasalocid (LAS), monensin (MON), and narasin (NAR) and their mixtures with cannabidiol (CBD) at a concentration of 1.56 µg/mL. Data are presented as mean ± SEM (*n* = 3).

Cell Line	Assay	Drugs							
		SAL	SAL + CBD	LAS	LAS + CBD	MON	MON + CBD	NAR	NAR + CBD
CRFK	MTT	14.9 ± 5.0 ^a^	24.9 ± 5.1 ^b^	28.8 ± 1.7 ^a^	27.5 ± 0.4 ^a^	>50	>50	>50	17.3 ± 3.2
	NRU	36.3 ± 6.3 ^a^	44.2 ± 10.3 ^b^	>50	>50	6.7 ± 0.7 ^a^	6.8 ± 0.3 ^a^	5.7 ± 0.2 ^a^	5.6 ± 0.2 ^a^
	TPC	>50	>50	>50	>50	>50	>50	>50	>50
	LDH	>50	>50	>50	>50	>50	>50	>50	>50
MDCK	MTT	>50	1.7 ± 0.5	7.7 ± 0.1 ^a^	7.8 ± 0.1 ^a^	>50	>50	7.1 ± 0.3 ^a^	49.5 ± 0.2 ^b^
	NRU	>50	3.5 ± 1.4	5.4 ± 0.2 ^a^	5.8 ± 0.5 ^a^	19.4 ± 1.1 ^a^	20.6 ± 5.1 ^a^	39.0 ± 3.6 ^a^	36.3 ± 5.3 ^a^
	TPC	>50	7.5 ± 0.6	47.0 ± 2.2 ^a^	6.8 ± 0.5 ^b^	>50	>50	>50	>50
	LDH	>50	>50	>50	>50	>50	>50	>50	>50

Different lowercase letters (a, b) within each row indicate statistically significant differences between the drug and its mixtures with CBD (*p* ≤ 0.05).

**Table 3:** Classification of CBD–coccidiostat interactions by cell line and assay endpoint. Interactions were classified from combination index values (Figure 2) as synergistic (CI < 0.9), additive (CI 0.9–1.1), or antagonistic (CI > 1.1). Where the classification varied with concentration, the ionophore concentrations (µg/mL) to which each applies are given in brackets.

	MTT	NRU	TPC	LDH
**CRFK**				
**SAL**	Synergistic (10, 50); Additive (1, 5)	Additive (1, 10, 50); Antagonistic (5)	Antagonistic	Additive (1–10); Synergistic (50)
**LAS**	Antagonistic (1–10); Additive (50)	Antagonistic	Antagonistic	Antagonistic
**MON**	Antagonistic	Additive	Antagonistic	Antagonistic
**NAR**	Antagonistic; Synergistic at high concentration (50)	Additive	Borderline additive/antagonistic	Antagonistic
**MDCK**				
**SAL**	Additive (1) Synergistic (5–50)	Synergistic	Synergistic	Synergistic
**LAS**	Additive (1); Synergistic (5–50)	Synergistic (1–10); Borderline synergistic/additive	Additive (1, 5); Synergistic (10, 50)	Borderline synergistic/additive (1); Synergistic (5–50)
**MON**	Antagonistic	Antagonistic (1, 5); Additive (10, 50)	Antagonistic	Additive
**NAR**	Antagonistic	Antagonistic (1, 5); Additive (10, 50)	Antagonistic	Additive

**Table 4:** Predicted binding affinities and interacting residues for cannabidiol, the four ionophore coccidiostats, and three reference ligands docked to human VDAC1. Values are the mean ± SD of the top-ranked pose across three independently seeded runs. HB, hydrogen bond; HI, hydrophobic interaction; DHA, hydrogen-to-acceptor distance; DA, donor-to-acceptor distance (Å).

Group	Ligand	Binding Affinity ΔG (kcal/mol)			Mean ± SD	Interacting Residues (Bond Length Å)
		Rep 1	Rep 2	Rep 3		
**Individual screening**	CBD	−6.97	−6.72	−6.67	**−6.79 ± 0.16**	**HB**: Ser234 (DHA 3.19, DA 3.80) **HI**: Val17 (3.82), Ala205 (3.70), Ala222 (3.45), Lys236 (3.83, 3.84), Leu262 (3.46)
	SAL	−7.76	−7.64	−7.97	**−7.79 ± 0.17**	**HB**: Arg15 (DHA 2.35, DA 2.80), Thr19 (DA 2.77), Gly21 (DHA 2.84, DA 3.40) **HI**: Leu26 (3.88), Lys28 (3.94, 3.36, 3.68), Ala222 (3.77), Thr258 (3.97)
	LAS	−8.20	−8.35	−8.08	**−8.21 ± 0.14**	**HB**: Lys236 (DHA 3.14, DA 3.56), Gly244 (DHA 2.52, DA 2.92), Gly246 (DHA 2.93, DA 3.32), Ser260 (DHA 2.87, DA 3.20) **HI**: Tyr22 (3.49), Leu26 (3.57), Lys28 (3.64), Ala222 (3.60), Lys236 (3.73, 3.63) **π interaction**: Lys236 (5.40)
	MON	−8.25	−7.65	−8.37	**−8.09 ± 0.39**	**HB**: Asp16 (DHA 2.36, DA 3.27), Ser46 (DHA 2.30, DA 3.15), Asn76 (DHA 2.31, DA 3.19), Ser234 (DHA 3.74, DA 4.08), Ser260 (DHA 2.37, DA 3.24) **HI**: Lys28 (3.88, 3.54), Thr55 (3.34), Lys236 (3.98, 3.65)
	NAR	−8.29	−8.37	−8.06	**−8.24 ± 0.16**	**HB**: Arg15 (DHA 2.70, DA 3.08), Thr19 (DHA 2.08, DA 3.00), Ser260 (DHA 2.00, DA 2.94) **HI**: Tyr22 (3.77), Leu26 (3.16), Lys28 (3.60, 3.56), Lys236 (3.50)
**Simultaneous co-docking**	CBD + SAL	−9.97	−10.31	−10.40	**−10.23 ± 0.23**	**CBD** **HB**: Asp16 (DHA 2.20, DA 3.12), Thr248 (DHA 3.44, DA 3.87) **HI**: Lys224 (3.77), Lys236 (3.80), Thr258 (3.96) **SAL** **HB**: Asp16 (DHA 3.38, DA 3.71), Lys28 (DHA 2.33, DA 3.13) **HI**: Arg15 (3.82), Thr19 (3.78), Tyr22 (3.65), Lys28 (3.47) **Salt bridges**: Lys28 (2.98)
	CBD + LAS	−10.60	−11.25	−11.08	**−10.98 ± 0.34**	**CBD** **HB**: Tyr22 (DHA 2.61, DA 2.97), Ser46 (DHA 2.01, DA 2.68) **HI**: Phe24 (3.59, 3.81, 3.57, 3.54), Leu26 (3.99), Lys53 (3.76) **LAS** **HB**: Asp16 (DHA 3.39, DA 3.24), Ser260 (DHA 2.23, DA 3.09), Leu275 (DHA 3.43, DA 3.94) **HI**: Leu26 (3.52), Lys28 (3.89), Ala222 (3.53), Lys236 (3.62), Thr258 (3.84) **π interaction**: Lys28 (4.17) **Salt bridges**: Lys28 (4.06)
	CBD + MON	−10.59	−10.88	−10.79	**−10.75 ± 0.15**	**CBD** **HB**: Ser260 (DHA 1.98, DA 2.90) **HI**: Phe24 (3.54), Thr258 (3.67) **MON** **HB**: Lys28 (DHA 2.55, DA 3.02), Ser46 (DHA 2.15, 3.26, DA 2.87, 3.70), Thr55 (DHA 2.64, 2.21, DA 3.27, 2.86) **HI**: Phe24 (3.53), Lys28 (3.80), Lys53 (3.46) **Salt bridges**: Arg15 (4.78)
	CBD + NAR	−9.43	−10.61	−9.78	**−9.94 ± 0.61**	**CBD** **HB**: Thr19 (DHA 2.57, DA 3.46), Ser260 (DHA 1.96, 2.06, DA 2.74, 2.74) **HI**: Thr19 (3.85), Ala222 (3.50), Lys236 (3.76) **NAR** **HB**: Lys28 (DHA 3.01, 2.42, DA 3.37, 3.37), Ser44 (DHA 2.56, DA 3.24), Ser46 (DHA 2.36, DA 2.83), Lys74 (DHA 3.13, DA 4.07) **Salt bridges**: Lys28 (3.93)
**Reference**	NADH	−9.11	−8.88	−8.81	**−8.93 ± 0.16**	**HB**: Gly21 (DHA 2.53, DA 3.38), Lys28 (DHA 2.31, DA 3.14), Ser234 (DHA 2.67, DA 3.20), Lys236 (DHA 3.25, DA 3.66), Leu245 (DHA 2.64, DA 3.60), Gly246 (DHA 3.23, DA 3.56), Thr248 (DHA 2.62, 2.68, DA 3.11, 3.16), Ser260 (DHA 2.81, DA 3.56), Ala261 (DHA 2.31, 2.43 DA 2.93, 3.04), Glu280 (DHA 2.20, DA 2.78) **HI**: Lys28 (3.76, 3.91), Thr258 (3.35) **Salt bridges**: Lys236 (5.32, 4.99)
	VBIT-4	−7.91	−8.47	−8.04	**−8.14 ± 0.29**	**HB**: Gly23 (DHA 3.00, DA 3.34), Ser260 (DHA 2.17, DA 3.03) **HI**: Tyr22 (3.89), Phe24 (3.98, 3.68), Leu26 (3.94), Lys236 (3.70, 3.86) **π interaction**: Lys236 (5.31)
	SW016789	−7.70	−7.77	−7.72	**−7.73 ± 0.04**	**HI**: Lys28 (3.74, 3.80), Lys236 (3.63), Thr258 (3.80) **π interaction**: Lys236 (5.63)
**Competitive displacement**	CBD + SAL + LAS + MON + NAR	−12.42	−12.71	−12.46	**−12.53 ± 0.16**	**CBD HI**: Arg93 (3.50) **SAL HB**: Asp16 (DHA 2.65, 2.67, DA 3.27, 3.34). **HI**: Lys12 (3.89), Arg15 (3.61), Thr19 (3.40) **LAS HB**: Lys119 (DHA 3.05, DA 3.61). **HI**: Met1 (3.52), Ala92 (3.89), Arg93 (1.87, 2.14), Leu95 (3.21) **MON HB**: Met1 (DHA 2.28, DA 2.96). **HI**: Met1 (2.47), Ala2 (3.14) **NAR HI**: Met1 (2.28), Ala2 (3.94), Tyr67 (3.51)
	SAL + LAS + MON + NAR	−11.75	−10.65	−12.06	**−11.49 ± 0.74**	**SAL HB**: Lys28 (DHA 2.71, 1.84, DA 3.15, 2.85). **HI**: Thr19 (3.51) **LAS HB**: Lys119 (DHA 3.32, DA 4.01). **HI**: Ala2 (3.75), Arg93 (1.45) **MON HB**: Met1 (DHA 3.04, DA 3.55). **HI**: Met1 (1.14), Val3 (3.71), Arg93 (3.75, 3.62) **NAR HB**: Arg93 (DHA 3.00, DA 3.68). **HI**: Met1 (2.13), Ala2 (3.06), Arg93 (3.28)
	CBD+NADH	−10.82	−10.40	−10.47	**−10.56 ± 0.23**	**CBD** **HB:** Thr55 (DHA 2.58, DA 3.18). **HI**: Tyr22 (3.96), Phe24 (3.65, 3.61, 3.75, 3.58), Leu26 (3.81), Lys53 (3.76), Thr55 (3.70) **NADH** **HB**: Asp16 (DHA 2.81, DA 3.26), Tyr22 (DHA 2.39, DA 3.01), Ser234 (DHA 2.62, DA 3.32), Lys236 (DHA 2.84, DA 3.25), Leu245 (DHA 2.84, DA 3.81), Gly246 (DHA 3.11, DA 3.54), Thr248 (DHA 2.33, DA 3.26), Ser260 (DHA 2.55, DA 3.28), Ala261 (DHA 2.53, 2.61, DA 3.19, 3.28) **HI**: Lys28 (3.43) **Salt bridges**: Lys236 (5.29, 4.77)

**Table 5:** Calculated physicochemical descriptors and docking-derived ligand efficiency for the individual compounds and multi-ligand conditions. Gap is the difference between the assembly score and the sum of its constituents, with positive values indicating that the assembly binds less favorably than its parts would separately. Ligand efficiency is reported here as a relative descriptor rather than a thermodynamic quantity. RotB, rotatable bond. TPSA, topological polar surface area. cLogP, calculated logarithm of the partition coefficient.

**Physicochemical Descriptors of the Docked Compounds**										
**Compound**	**Formula**	**MW (g/mol)**	**cLogP**	**TPSA (Å^2^)**	**HBD**	**HBA**	**RotB**	**Torsions**	**Rings**	**Heavy Atoms**
**CBD**	C_21_H_30_O_2_	314.5	5.85	40.5	2	2	6	8	2	23
**SAL**	C_42_H_70_O_11_	751.0	6.19	161.2	4	10	12	16	5	53
**LAS**	C_34_H_54_O_8_	590.8	5.84	133.5	4	7	13	17	3	42
**MON**	C_36_H_62_O_11_	670.9	4.27	153.4	4	10	10	14	5	47
**NAR**	C_43_H_72_O_11_	765.0	6.43	161.2	4	10	12	16	5	54
**β-NADH**	C_21_H_29_N_7_O_14_P_2_	665.4	−2.69	317.6	8	17	11	18	5	44
**VBIT-4**	C_21_H_23_ClF_3_N_3_O_3_	457.9	3.75	65.0	2	5	7	9	3	31
**SW016789**	C_20_H_19_N_3_OS	349.5	3.77	53.9	2	3	5	6	4	25
**Ligand Efficiency LE = −ΔG/Heavy Atoms (Relative Descriptor, Not Thermodynamic)**										
**Condition**	**ΔG**	**Heavy Atoms**	**Torsions**	**LE**	**Sum (Individual)**	**Gap**				
**CBD**	−6.79	23	8	0.295	-	-				
**SAL**	−7.79	53	16	0.147	-	-				
**LAS**	−8.21	42	17	0.195	-	-				
**MON**	−8.09	47	14	0.172	-	-				
**NAR**	−8.24	54	16	0.153	-	-				
**β-NADH**	−8.93	44	18	0.203	-	-				
**VBIT-4**	−8.14	31	9	0.263	-	-				
**SW016789**	−7.73	25	6	0.309	-	-				
**CBD + SAL**	−10.23	76	24	0.135	−14.58	4.35				
**CBD + LAS**	−10.98	65	25	0.169	−15.00	4.02				
**CBD + MON**	−10.75	70	22	0.154	−14.88	4.13				
**CBD + NAR**	−9.94	77	24	0.129	−15.03	5.09				
**CBD + β-NADH**	−10.56	67	26	0.158	−15.72	5.16				
**SAL + LAS + MON + NAR**	−11.49	196	63	0.059	−32.33	20.84				
**CBD + SAL + LAS + MON + NAR**	−12.53	219	71	0.057	−39.12	26.59				

## Data Availability

The data presented in this study are available upon request from the corresponding author.

## Supplementary Materials

The following supporting information can be downloaded at: https://www.mdpi.com/article/10.3390/molecules31213810/s1. Figure S1: The effect of cannabidiol (CBD) at concentrations of 1.56–12.5 µg/mL is shown in two kidney cell lines from cat (CRFK) and dog (MDCK). Data are presented as mean ± SD (standard deviation), *n* = 3. NC—negative control; PC—positive control. * *p* ≤ 0.05, ** *p* ≤ 0.01, *** *p* ≤ 0.001, **** *p* ≤ 0.0001 versus the negative control (one-way ANOVA with Dunnett’s post hoc test).
