# Supplementary material for: Cytotoxic Interactions Between Cannabidiol and Ionophore Coccidiostats in Feline and Canine Kidney Cell Lines—An In Vitro Study
Source: Molecules. 2026 Oct 22;31(21):3810. doi: 10.3390/molecules31213810 (PMC13764792; doi:10.3390/molecules31213810)
Supplement: Supplementary file 1 [file molecules-31-03810-s001.zip › molecules-4577912-supplementary.pdf]

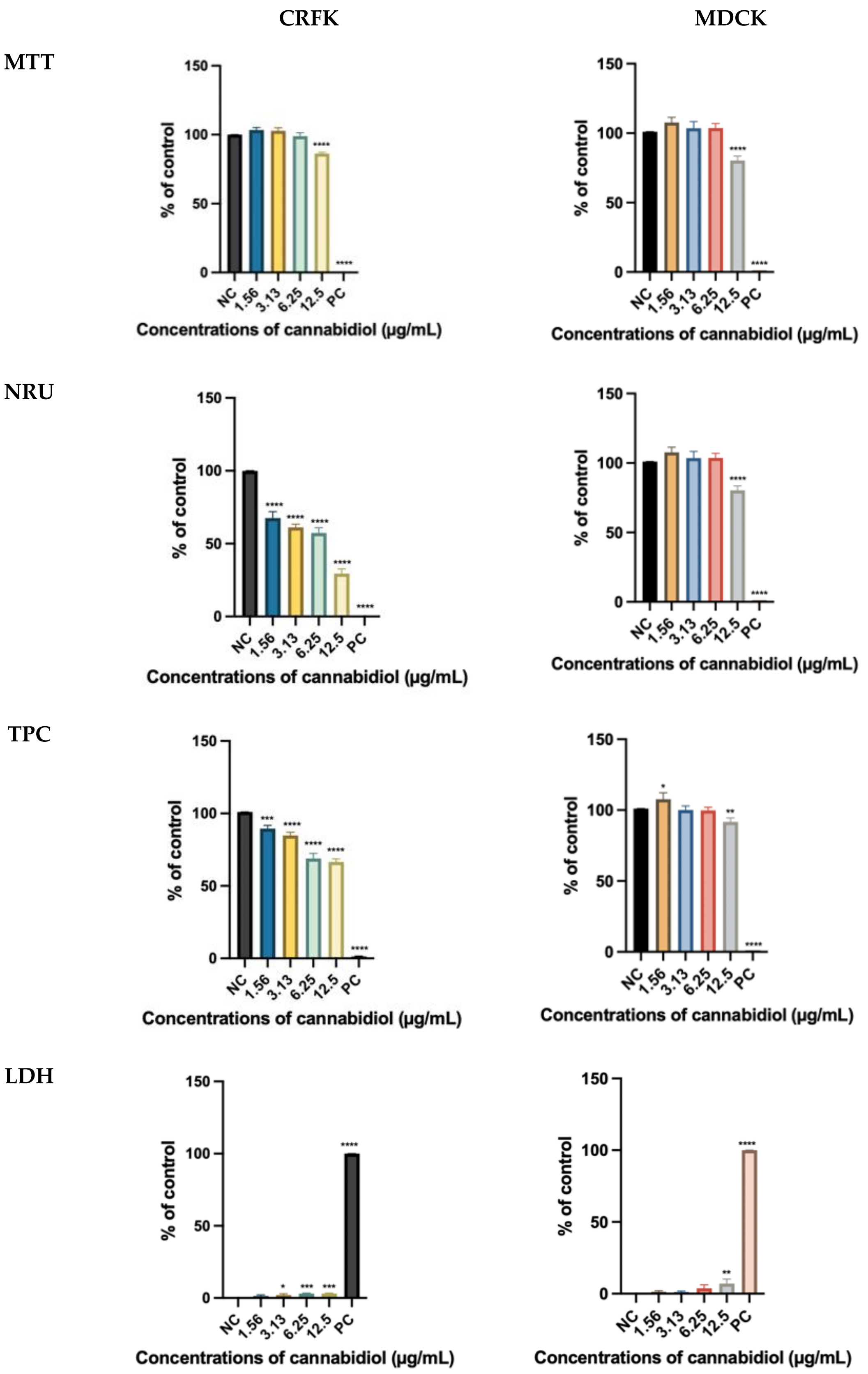

Figure S 1. The effect of cannabidiol (CBD) at concentrations of 1.56–12.5 µg/mL is shown in two kidney cell lines from cat (CRFK) and dog (MDCK). Data are presented as mean ± SD (standard deviation), n = 3. NC — negative control; PC — positive control. * $p \leq 0.05$, ** $p \leq 0.01$, *** $p \leq 0.001$, **** $p \leq 0.0001$ versus the negative control (one-way ANOVA with Dunnett's post hoc test).
